# Plant Growth-Promoting Microorganisms Mediate Plant Metabolic Reprogramming to Manage the Rhizospheric Microbiome

**DOI:** 10.3390/microorganisms14030578

**Published:** 2026-03-03

**Authors:** Pei Song, Yue Deng, Yaoying Yu, Lei Zhang, Yong Liu

**Affiliations:** 1 The College of Life Science, Sichuan University, Chengdu 610000, China; 2 Institute of Plant Protection, Sichuan Academy of Agricultural Sciences, Chengdu 610000, China; yuedeng@scsaas.cn (Y.D.); kellyyzsj@163.com (Y.Y.); zhanglei9296@aliyun.com (L.Z.); 3 Key Laboratory of Integrated Pest Management on Crops in Southwest, Ministry of Agriculture and Rural Affairs, Chengdu 610000, China

**Keywords:** root exudates, rhizosphere microbiome, phytohormone signaling, sustainable agriculture, multi-omics

## Abstract

The microbial community surrounding plant roots plays a vital role in plant growth, nutrient uptake, stress resilience and other potential functions. This review synthesizes available evidence that plant growth-promoting microorganisms (PGPMs) not only directly benefit the plant but also modulate the rhizospheric microbiome by mediating metabolic reprogramming of the host plant. PGPMs modify the composition of root exudates through the regulation of phytohormone signaling and transcriptional networks, thereby promoting beneficial microbes and suppressing disease. Key mechanisms involve the jasmonate, ethylene, and strigolactones signaling pathways. Transcription factors MYB72, ERF1 regulate biosynthesis and secretion of metabolites like organic acids and coumarins. The exudates serve as specific signals for microbial community assembly and as enhancers of feedback loops that reinforce plant-microbe mutualism. We examine the ecological and agricultural significance of PGPM-induced metabolic reprogramming of the host due to PGPMs that enhances disease suppression, abiotic stress tolerance, and nutrient use efficiency. Lastly, we address advanced methods and strategies for transferring these biological pathways to the agricultural realm and on to a more sustainable agricultural practice with emphasis on the need to integrate multi-omics (whole genomics, transcriptomics, and metabolomics), synthetic microbial communities and plant genetic engineering for microbiome-assisted agriculture. This synthesis reveals that PGPM-induced metabolic reprogramming operates through an integrated cross-scale framework linking microbial perception, phytohormone signaling, transcriptional regulators, and transporter-mediated exudate efflux, with root exudates functioning as plant-controlled ecological filters that selectively shape the rhizosphere microbiome. We further identify key translational challenges, including context-dependent efficacy and the lab-to-field gap, and propose a roadmap combining multi-omics, synthetic communities, and genome editing to realize the potential of microbiome-assisted sustainable agriculture.

## 1. Introduction

The interaction between plants and soil microorganisms is a major factor regulating plant growth, development, health and stress resistance in terrestrial ecosystems. Consequently, the rhizosphere (the soil microenvironment influenced by plant roots) represents a key ecological hotspot. Lorenz Hiltner was the first to conceptualize this interface in 1904. This interface is also recognized for its strong substance exchanges and signaling between roots, microbes, and soil components [1,2,3,4]. Just as the rhizosphere is critical for plant roots, the phycosphere, the immediate microenvironment surrounding microalgae, represents a similar hotspot for algae-bacteria interactions, underscoring the universal importance of such interfacial zones in shaping microbial consortia. This interface’s distinctiveness arises from exceptional microbial density and activity, primarily fueled by root-derived exudates that provide essential carbon and energy substrates [5,6,7]. In addition to possessing a high microbial density, the rhizosphere has a vast diversity of microorganisms and functional complexity, where relationships range from mutualism to parasitism, collectively influencing plant fitness [8]. Root-secreted metabolites act as the main mediators structuring microbial inhibitory community assembly and functions [6,9].

The rhizosphere microbiome is formed by the microbes colonizing the root surface and its surrounding area. The rhizosphere microbiome, which consists of bacteria, fungi, archaea, protists, microalgae, and viruses, is organised non-randomly. It is increasingly regarded as a functional extension of the plant host, acting as a ‘second genome’ that is essential for fitness, strength, and stress tolerance [10,11]. These microbes are not passive inhabitants but active agents that perform critical functions, such as: (1) Nutrient acquisition and cycling. Microbes enhance nutrient bioavailability through processes including mineralization (e.g., rhizobial nitrogen fixation [12,13]), solubilization (e.g., of phosphorus by phosphate-solubilizing bacteria [14]), and mobilization (e.g., of iron via siderophores [15]). (2) Pathogen suppression and immune modulation. As a first line of defense, the microbiome suppresses pathogens through niche competition, antibiosis, production of lytic enzymes, and iron competition via siderophores [16,17]. Furthermore, specific microbial taxa can induce systemic resistance (ISR) in the host plant, a mechanism that underpins the phenomenon of naturally disease-suppressive soils [18]. (3) Abiotic stress mitigation. Microbial enhancement of plant tolerance to drought, salinity, and heavy metal stress is achieved by: improving soil water retention via exopolysaccharide (EPS) production; synthesizing osmolytes and phytoprotectants; modulating ethylene signaling through enzymes like ACC deaminase; and boosting the plant’s antioxidant capacity [19,20,21,22]. (4) Growth regulation. Beyond their essential nutritional roles, microbes exert direct effects on plant development via the synthesis of phytohormones (e.g., auxins, cytokinins, and gibberellins) and by modulating root architecture, thereby influencing overall growth [23,24].

The positive influence of this microbial consortium is significantly mediated by plant growth-promoting microorganisms (PGPMs). PGPMs encompass a phylogenetically diverse group, including well-studied bacteria such as *Pseudomonas*, *Bacillus*, *Azospirillum*, *Rhizobium*, *Burkholderia* spp. and *Chlamydomonas reinhardtii* [25,26], beneficial fungi like arbuscular mycorrhizal fungi (AMF; e.g., *Rhizophagus* and *Funneliformis*) and *Trichoderma* [27,28], and emerging key players such as microalgae [29,30]. Other important PGPMs include certain archaea and cyanobacteria [31]. These beneficial microbes are not merely passive inhabitants but are increasingly recognized as central actors that actively reprogram host plant metabolism [32]. PGPMs exert beneficial effects by regulating the transcription and metabolism of host plants, such as responding to stress, improving nutrient utilization, and plant hormone synthesis (such as microbial auxins) [30,33]. A major research frontier now lies in understanding the specific mechanisms by which PGPMs shape the assembly and dynamics of the surrounding rhizosphere community through this reprogramming of the host. By modulating root exudation patterns from within the plant, PGPMs act as managers of the rhizosphere environment. This leads to the selective enrichment of beneficial consortia and the suppression of harmful taxa, thereby shaping a microbiome structure and function that ultimately benefits the plant. Plant health depends on a functional rhizosphere microbiome, whose disruption (dysbiosis) compromises nutrient uptake, disease resistance, and stress tolerance [34,35]. These microbial communities are assembled dynamically and non-randomly, co-evolving with the plant under environmental influence [12]. A central question is how plants steer this assembly via root exudate signaling to recruit and maintain a beneficial microbiota.

While the pivotal role of PGPMs in direct plant promotion is well established, existing reviews often address either the microbial partners, the plant’s signaling pathways, or the resulting microbiome composition in isolation. This review aims to consolidate current understanding by providing a cross-scale synthesis that explicitly links these levels. We focus on elucidating the integrated molecular mechanisms—from the initial phytohormone signaling (e.g., jasmonic acid, ethylene, strigolactones) and activation of key transcription factors to the consequent transcriptional reprogramming of metabolic pathways and transporter genes. This framework directly connects these plant-autonomous processes to the altered root exudate profiles that selectively assemble beneficial microbial consortia. By integrating these layers of regulation, this review offers a novel and systematic perspective on how PGPMs mediate host metabolism to manage the rhizosphere microbiome, an aspect not comprehensively integrated in the previous literature. We further discuss the ecological and agronomic implications of this reprogramming and highlight methodological advances and persisting challenges, thereby providing a coherent framework for future research toward microbiome-assisted sustainable agriculture. Table 1 provides representative PGPM groups, their functions, and documented case studies.

## 2. Root Exudates: The Currency of Root Microenvironment Exchange

**Figure 1:**
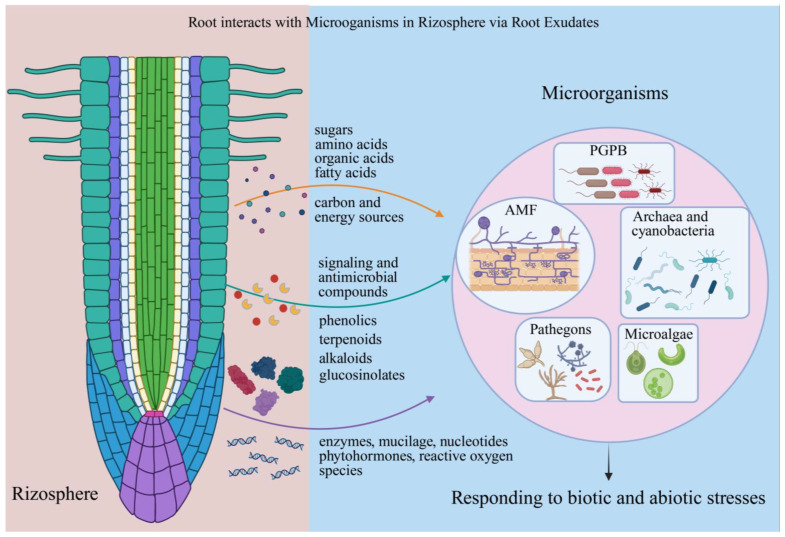
Root-microbe interactions in the rhizosphere mediated by root exudates. Root exudates comprise primary metabolites (sugars, amino acids, organic acids, fatty acids) and secondary metabolites (flavonoids, coumarins, strigolactones et al.). These compounds function as signaling molecules (e.g., flavonoids attract rhizobia; strigolactones recruit AMF), nutrient mobilizers (e.g., organic acids solubilize phosphate; coumarins chelate iron), and antimicrobial agents (e.g., phytoalexins inhibit pathogens). PGPB: plant growth-promoting bacteria; AMF: arbuscular mycorrhizal fungi. Created with BioRender (www.biorender.com).

Plants shape their rhizosphere microbiome primarily through root exudates. These exudates function as metabolic substrates, signaling molecules, or modifiers of the physico-chemical environment (Figure 1). Primary metabolites, such as sugars (e.g., glucose, sucrose), amino acids, organic acids (e.g., citrate, malate), and fatty acids, provide essential carbon and energy to fuel microbial growth and activity [44]. Beyond primary metabolites, roots also release a diverse array of specialized secondary metabolites. These include, for example, phenolic compounds (flavonoids, coumarins), terpenoids (e.g., strigolactones), alkaloids, and glucosinolates [45]. In addition to their role as signals and antimicrobials, these metabolites are part of a broader exudate profile that includes functional proteins (e.g., enzymes), structural polymers like mucilage, nucleotides, phytohormones, and reactive oxygen species (ROS) [46,47]. Together, this collection is a sophisticated biochemical language that structures and defines plant manipulation of microbes.

The rhizosphere microbiome is coordinated by root exudates through multiple interconnected mechanisms. Root exudates provide essential nutrients that support microbial colonization, with organic acids and sugars serving as preferred carbon sources. Their selective utilization helps stabilize specific microbial populations and establishes the foundational nutritional framework of the community [48]. Furthermore, root exudates act as signaling mediators between plants and microorganisms. Crucially, these signaling molecules are not static; instead, they are dynamically modulated as a direct consequence of the plant’s perception of rhizosphere microbes and the subsequent metabolic reprogramming induced by them. For example, flavonoids function as specific chemical inducers of nodulation in rhizobia, while strigolactones (SLs) promote hyphal branching and colonization by AMF [49,50]. Plants can also interfere with potential pathogens by secreting metabolites that mimic or antagonize bacterial quorum-sensing signals, thereby disrupting the production of virulence factors, biofilm formation, and other group behaviors [51]. In response to pathogen recognition, plants release antimicrobial compounds such as phytoalexins, specific phenolics, and benzoxazinoids, which directly inhibit pathogenic activity [52]. Thus, the exudate-mediated chemical dialogue in the rhizosphere reflects the dynamic, reprogrammed metabolic state of the plant, shaped by its ongoing interactions with the microbial community. Additionally, organic acids and phytosiderophores exuded by roots can chelate metal ions (e.g., Fe^3+^, Al^3+^) and solubilize mineral nutrients (e.g., phosphorus, potassium), thereby altering local pH and nutrient bioavailability [46]. This chemically modified microenvironment selectively favors or inhibits different microbial taxa. Under these conditions, microorganisms that contribute to plant nutrient acquisition, stress tolerance, or growth promotion become enriched. This selective enrichment is not a passive outcome but an active process: root exudates function as a dynamic, plant-controlled ecological filter. The selectivity of this filter—the specific blend of metabolites secreted into the rhizosphere—is actively shaped by PGPM-induced metabolic reprogramming, as detailed in the following sections. Through this mechanism, plants, in partnership with beneficial microbes, cultivate a rhizosphere microbiome that ultimately supports plant health and fitness.

## 3. Rhizosphere Microbes-Mediated Plant Metabolic Reprogramming

Plant metabolic reprogramming is a key adaptive strategy, defined as a systemic and coordinated shift in the plant’s transcriptional and metabolic state triggered by environmental cues, such as rhizosphere microbial colonization. It is important to distinguish direct PGPM effects (immediate microbial actions) from metabolic reprogramming (systemic, persistent plant changes triggered by microbial perception). Direct effects refer to the direct actions mediated by microbial molecules themselves, such as plant hormone production (such as microbial IAA), nutrient dissolution (such as organic acid dissolution of phosphate), or pathogen inhibition (such as antibiotic production), which occur independently of sustained changes in plant metabolism. In contrast, metabolic reprogramming involves systematic and persistent changes in plant transcription and metabolic status induced by microbial perception, resulting in persistent changes in root exudate composition and plant physiology. This process redirects metabolic fluxes from primary to specific secondary pathways, such as flavonoid or coumarin biosynthesis, to enhance growth and defense [53]. This reprogramming extends beyond localized responses to involve transcriptional rewiring of metabolic genes and modulation of enzyme activities. Mechanistic studies have confirmed that PGPMs can activate crucial transcriptional regulators like MYB72, which orchestrates systemic changes in root exudate profiles [54,55]. A concrete example is observed in tomato, where rhizosphere microbes were shown to directly alter azelaic acid hexose concentrations and drive specific metabolic reprogramming via the systemically induced root exudation of metabolites (SIREM) process [6]. Such reprogramming has been linked to dynamic adjustments in exudate composition—for instance, the induction of transporters such as ALMT1 (aluminum-activated malate transporter) has been linked to facilitating organic acid efflux, establishing feedback loops that may enhance plant resilience under stress [9,48]. Under salinity stress, PGPM-primed plants have been observed to exhibit metabolic plasticity, sustaining rhizosphere pH through malate secretion and correlating with significantly boosted antioxidant capacity of the associated microbial community [46].

PGPM regulates plant metabolic reprogramming (Table 2) through an integrated plant hormone signaling network, particularly signaling pathways such as jasmonic acid (JA), ethylene (ET), and SLs, which focus on key transcriptional regulatory factors and activate the expression of related genes. Upon perception of beneficial bacteria and fungi, the JA and ethylene ET signaling pathways converge to activate key transcription factors such as MYB72 and ERF1. These regulators in turn induce the biosynthesis of defense-related and signaling metabolites [55]. For instance, certain *Pseudomonas* species elicit induced systemic resistance (ISR) largely via JA/ET-dependent pathways, resulting in increased production of antimicrobial compounds [18]. Similarly, GRAS-domain transcription factors can stimulate SLs secretion, which promotes hyphal colonization by AMF and facilitates subsequent nutrient exchange with the host plant [56]. Collectively, these signaling cascades illustrate how PGPMs act as biological triggers that reshape plant metabolism. The resulting metabolic shifts contribute to the assembly of a protective and nutritionally efficient rhizosphere microbiome.

Root exudates function as both outputs and regulators of the metabolic reprogramming driven by PGPMs, establishing feedback loops that reinforce plant–microbe mutualism. Secreted metabolites-such as organic acids, coumarins, and SLs-alter soil biochemistry and microbial activity, thereby modulating the composition and function of the rhizosphere microbiome. For example, coumarins can chelate rhizosphere iron, inducing an iron-deficiency response that further upregulates coumarin biosynthesis through FIT (fer-like iron-deficiency induced transcription factor)-dependent signaling [5]. Similarly, organic acids (e.g., citrate) acidify the rhizosphere, which increases phosphate solubility and activates plant phosphate-starvation responses, leading to enhanced expression of genes encoding organic acid transporters [48]. These mechanisms are ecologically significant because under stress, microbe-induced metabolite release helps maintain microbial fitness and restores microbiome equilibrium.

The ecological significance of PGPM-induced metabolic reprogramming lies in its ability to enhance plant fitness by selectively shaping the rhizosphere microbiome. Through the modulation of root exudate profiles, PGPMs promote the enrichment of beneficial microbial taxa, such as *Streptomyces* and AMF. These enriched microbes support plant health by facilitating nutrient acquisition, suppressing pathogens, improving abiotic stress tolerance, and promoting growth [7,20]. For example, the secretion of coumarins can recruit siderophore-producing bacteria, which competitively exclude fungal pathogens [57,58]. Furthermore, under drought conditions, PGPM inoculation has been shown to engage both the root-associated co-occurring microbial community and the soil nitrogen-fixing community through exudate-mediated networks [38,59]. Thus, metabolic reprogramming represents a key plant-mediated mechanism for maintaining ecosystem function. Consequently, strategies leveraging PGPMs provide a sustainable approach to enhancing crop resilience and productivity.

**Table 2:** Representative examples of PGPM-induced metabolic reprogramming in plants.

Plant Species	PGPM Strain	PGPM Type	Key Metabolic Changes	Function	References
*Brassica napus* L.	*Bacillus amyloliquefaciens*	Bacteria	flavonoid and anthocyanin biosynthesis ↑	Plant growth-promoting	[53]
*A. thaliana*	*P. simiae*	Bacteria	MYB72/FRO2/IRT1/FIT ↑	induced systemic resistance, iron acquisition	[54]
*A. thaliana*	*P. simiae*, *P. capeferrum*	Bacteria	MYB72/BGLU42 ↑, scopoletin ↑	improved niche establishment for the microbial partner	[55]
*A. thaliana*	*B. amyloliquefaciens*, *T. harzianum*	Bacteria, Fungi	SA and JA/ET signaling pathways activation	induced systemic resistance	[60,61]
*A. thaliana*	*T. harzianum*	Fungi	*MYB72* expression ↑, NO accumulation	ISR against *Botrytis cinerea*	[62]
*Solanum lycopersicum* (tomato)	Rhizosphere microbiome (undefined)	Microbial consortium	Azelaic acid hexose ↑, SIREM activation	Systemic metabolic reprogramming, stress resilience	[6]
*Triticum aestivum* (wheat)	*Bacillus velezensis*	Bacteria	Proline ↑, GABA ↑, flavonoids ↑	Drought stress tolerance	[63]
*Oryza sativa* (rice)	*Chlorella vulgaris*, N_2_-fixing bacteria	Microalgae-bacteria consortium	Biomass ↑, N content ↑	Enhanced N availability, improved germination	[64]
*Glycine max* (soybean)	*Bradyrhizobium japonicum*	Bacteria	Flavonoid exudation ↑, nodulation ↑	N_2_ fixation enhancement	[65]
*Cucumis sativus* (cucumber)	PGPR consortium	Bacteria	Phenolic acids ↑, flavonoids ↑	Resistance against *Fusarium oxysporum*	[66]
*Chlamydomonas reinhardtii*	*Methylobacterium aquaticum*	Alga, Bacteria	IAA (from alga) ↑, IAA (degraded by bacterium) ↓	Mutualistic growth promotion	

Note: “↑” represents “increase”, “↓” represents “decrease”.

## 4. Molecular Mechanisms of PGPMs-Induced Metabolic Reprogramming

### 4.1. Phytohormone Signaling: The Central Orchestrator

**Figure 2:**
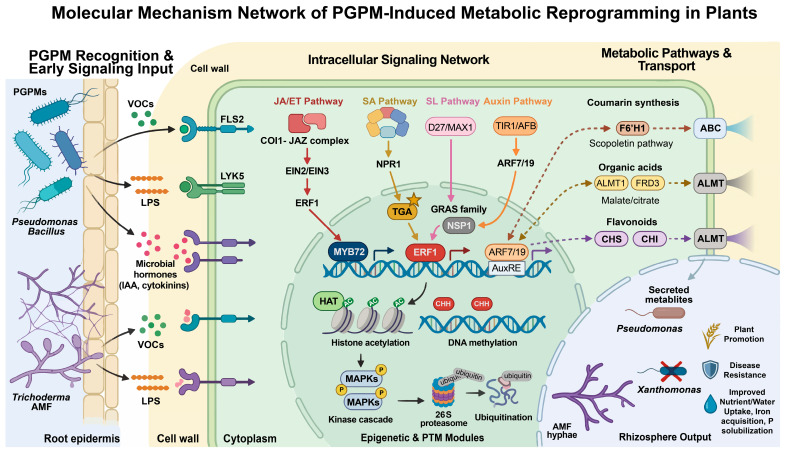
Molecular mechanisms of PGPM-induced metabolic reprogramming in plant roots. Microbial signals such as LPS and VOCs are perceived by plant root cells, activating MAPK cascades. The activated MAPKs modulate phytohormone signaling pathways, including JA, ET, SL and auxin. Key transcription factors such as MYB72 and ERF1 are induced through JA/ET signaling. These transcription factors coordinate the expression of biosynthetic genes (e.g., CHS, CHI for flavonoids; F6’H1 for coumarins) and transporter genes (e.g., ABC transporters, ALMT1). The resulting metabolites, including coumarins (e.g., scopoletin), organic acids, and flavonoids, are secreted into the rhizosphere, where they shape the microbial community by suppressing pathogens and enriching beneficial taxa. LPS: lipopolysaccharide; COI1: F-box protein coronatine insensitive1; JAZ: Jasmonate zim-domain; CHS: Chalcone synthase; MAPK: mitogen-activated protein kinase; CHI: Chalcone isomerase.

PGPMs initiate host metabolic reprogramming largely through the modulation of phytohormone signaling networks (Figure 2). A key mechanism is the induction of systemic resistance. Microbial molecules serve as external triggers: for example, beneficial bacteria including certain *Pseudomonas* and *Bacillus* species release volatile organic compounds (VOCs) or lipopolysaccharides that are perceived by the plant [18,67]. In response, plant hormonal pathways mediated by JA, ET, and salicylic acid (SA) function as internal integration hubs, transducing these microbial signals into coordinated defense responses. For instance, VOCs produced by endophytic bacteria such as *Pantoea* sp. dez632 and *Pseudomonas* sp. bt851 have been shown to upregulate defense-related genes in sugar beet, including the soft-rot resistance gene *NBS-LRR2* and the pathogenesis-related gene *PR1*, indicating the involvement of JA and SA pathways, respectively, in this induced resistance [68]. Another major hormonal axis involves microbially derived auxins and cytokinins. For example, *Methylobacterium* sp. 2A synthesizes indole-3-acetic acid (IAA), enhances auxin responses in roots, and can restore lateral root formation and gravitropism in auxin-deficient mutants [69]. A recent study in the green alga *C. reinhardtii* revealed a similar IAA-dependent mutualism: under nitrogen limitation, the algal enzyme L-amino acid oxidase 1 (LAO1) produces IAA from tryptophan, which inhibits algal proliferation. Co-culture with *Methylobacterium* aquaticum degrades this IAA, relieving inhibition and promoting growth of both organisms—an interaction dependent on algal IAA production and bacterial IAA degradation [41]. This finding underscores the conserved role of auxin as an interkingdom signal in both the rhizosphere and phycosphere. Similarly, *P. chlororaphis* subsp. *Aurantiaca* promotes root branching by elevating auxin accumulation in lateral root primordia via an ARF7/19-dependent pathway [70]. Such microbial auxins integrate into the host’s signaling networks to reshape root architecture and exudation patterns. Microbially produced cytokinins can further alter source-sink relations, promoting carbon allocation to roots and enhancing root exudation [71,72]. In the context of AMF symbiosis, plant recognition of microbes upregulates SLs biosynthesis through transcription factors such as D27 and MAX1 [73,74,75]. These SLs not only promote hyphal branching of AMF but also act as signals recruiting other beneficial microbes to the rhizosphere.

### 4.2. Transcriptional Reprogramming: Key Regulators and Their Targets

PGPM-mediated modulation of plant gene expression is largely orchestrated through key transcription factors. Table 3 summarizes key transcriptional regulators and transporters involved in PGPM-induced metabolic reprogramming, including their types, inducing signals, target processes, and functions in the rhizosphere. A well-characterized example is MYB72, which in *Arabidopsis* and other plants is activated by PGPMs via JA/ET signaling. MYB72 regulates genes involved in iron mobilization and coumarin biosynthesis, including FRD3 (ferric reductase defective 3) and scopoletin 8-hydroxylase. The subsequent secretion of coumarins such as scopoletin shapes the rhizosphere microbiome by suppressing pathogens and enriching beneficial taxa [55,76,77]. Another integrative regulator is ERF1, which converges JA and ET signals to upregulate defense-related genes, particularly those encoding antimicrobial proteins and enzymes involved in secondary metabolism [78]. In the context of symbiosis, GRAS-domain transcription factors—such as NSP1 and NSP2, which are influenced by arbuscular mycorrhizal fungi—govern SLs biosynthesis and symbiosis-related genes. These factors also modulate transporter expression to facilitate bidirectional nutrient exchange between the host and its microbial partners [79,80].

Importantly, these transcriptional responses are fine-tuned in space and time. For instance, *MYB72* expression is rapidly induced in root epidermal and cortical cells upon perception of microbial signals such as volatile compounds from *Trichoderma* spp. or rhizobacterial elicitors [40,54], but its expression attenuates over time [55,62]. This spatial restriction to the root periphery aligns with the site of initial microbe-plant contact and subsequent metabolic reprogramming for exudate production. Moreover, different PGPMs may recruit overlapping yet distinct transcriptional modules: while *Pseudomonas* spp. strongly activate MYB72 and downstream coumarin biosynthetic genes (e.g., F6’H1) [55], beneficial fungi like *Trichoderma* preferentially engage MYB72 via distinct upstream signals involving nitric oxide [62] and also modulate GRAS-family regulators during symbiosis [80]. Furthermore, bacteria can rapidly evolve enhanced mutualism, with adaptations such as increased tolerance to MYB72-regulated scopoletin and mutations in the GacS/GacA system, leading to stronger MYB72 induction and plant growth promotion [81]. This specificity suggests that plants possess a suite of transcriptional programs that can be differentially deployed depending on the microbial partner, enabling tailored metabolic responses to diverse rhizosphere associates.

### 4.3. Epigenetic and Post-Translational Regulation

Beyond transcriptional regulation, metabolic reprogramming is fine-tuned through epigenetic and post-translational mechanisms. Microbial colonization can induce epigenetic modifications, such as DNA methylation and histone acetylation, which prime plant defense and metabolic responses. For instance, histone acetylation at defense gene promoters can sustain their expression beyond the initial microbial stimulus [82,83]. At the post-translational level, kinase cascades activated by microbe-associated molecular patterns (MAMPs) phosphorylate key metabolic enzymes to modulate their activity. Concurrently, ubiquitin-mediated degradation of transcriptional repressors derepresses metabolic genes, enabling rapid and precise adjustments to the plant’s metabolic state [84].

Among these post-translational mechanisms, mitogen-activated protein kinase (MAPK) cascades serve as central signaling hubs that integrate diverse environmental cues, including microbial signals and nutrient availability. Systematic analysis in the green alga *C. reinhardtii* identified 17 MAPKs, 2 MAPKKs, and 108 MAPKKKs, revealing the complexity of this signaling network in photosynthetic organisms [85]. Notably, the RAF-type MAPKKKs RAF14 and RAF79 showed strong transcriptional repression by ammonium, independent of the master nitrogen regulator NIT2, suggesting their potential role as nitrogen signal sensors [85]. This finding links MAPK signaling to nitrogen metabolism—a connection further supported by studies in *Arabidopsis* where MAPK6 phosphorylates nitrate reductase to modulate nitric oxide (NO) production [86]. Given that nitrogen availability profoundly influences root exudation and rhizosphere interactions, these MAPK components may represent important nodes where nutritional status and microbial signals are integrated to coordinate PGPM-induced metabolic reprogramming.

### 4.4. Regulation of Transporters: Gatekeepers of Exudation

The efflux of reprogrammed metabolites into the rhizosphere represents a critical, transporter-mediated endpoint of microbial-induced metabolic shifts. This process is largely governed by the induction of specific transporter families. Aluminum-activated malate transporters (ALMTs), for instance, are upregulated following PGPM (e.g., *Paenibacillus*, *Lysinibacillus*, *Burkholderia*, and *Bacillus*) colonization or under abiotic stress, facilitating the secretion of organic acids such as malate and citrate. This exudation serves a dual role: acidifying the rhizosphere to improve phosphate solubility and providing a carbon substrate for beneficial microbes [87,88,89]. Similarly, ATP-binding cassette (ABC) transporters are essential for the secretion of diverse secondary metabolites, including flavonoids, coumarins, and terpenoids. Their expression is frequently enhanced by PGPMs (e.g., *Microbacterium ginsengiterrae* and *Enterobacter asburiae*) and is vital for sustaining the flux of these bioactive compounds into the soil environment [90,91].

## 5. Application Advantages of PGPMs Mediated Metabolic Reprogramming

### 5.1. Shaping a Disease-Suppressive Microbiome

PGPM-induced exudation of specific metabolites allows plants to selectively recruit beneficial microorganisms, thereby shaping a protective rhizosphere microbiome. For instance, the targeted secretion of benzoxazinoids in maize enriches populations of *Pseudomonas* species, which suppress pathogens through antibiotic production and niche competition [65]. In cucumber, PGPM priming enhances the release of phenolic acids and flavonoids, contributing to induced resistance against *Fusarium oxysporum* [66]. This metabolite-mediated recruitment of beneficial taxa highlights the potential of PGPMs to support the development of naturally suppressive soils, offering a sustainable strategy to reduce dependence on synthetic pesticides.

### 5.2. Enhancing Abiotic Stress Tolerance

Through metabolic reprogramming, PGPMs strengthen plant resilience to abiotic stresses while supporting microbial function and soil health [21]. Under drought, PGPM-inoculated plants sustain the secretion of osmolytes and exopolysaccharides, which enhance soil water retention and promote microbial survival [92,93,94]. In saline conditions, PGPMs stimulate organic acid exudation to mitigate sodium toxicity by regulating ion homeostasis [95]. Additionally, ACC deaminase activity in PGPMs reduces ethylene-mediated stress, thereby encouraging root growth under adverse environments [96,97]. Collectively, these adjustments demonstrate how metabolite-driven feedback loops sustain plant–microbe interactions during stress.

### 5.3. Improving Nutrient Acquisition and Use Efficiency

PGPMs can enhance nutrient bioavailability by reprogramming root exudate composition, although the causal link between altered exudation and improved nutrition varies across mechanisms. Direct evidence links exudate changes to nutrient acquisition in several cases. Under iron limitation, PGPM-induced secretion of coumarins such as scopoletin chelates ferric iron (Fe^3+^), increasing its solubility and uptake through a process directly mediated by root exudate composition [98,99]. Similarly, under phosphorus limitation, enhanced exudation of organic acids including citrate and malate acidifies the rhizosphere and solubilizes insoluble phosphates, directly improving P availability. In legumes, PGPM-mediated flavonoid exudation acts as a signaling molecule that directly facilitates rhizobial nodulation and subsequent nitrogen fixation [100]. Beyond this well-established legume-rhizobia symbiosis, emerging research highlights the biotechnological potential of microalgae and nitrogen-fixing bacterial consortia. These associations, occurring naturally in the phycosphere, can be harnessed to enhance nitrogen availability in non-legume crops through complementary mechanisms: microalgae provide fixed carbon and oxygen, while diazotrophic bacteria supply biologically available nitrogen [101]. Experimental evidence demonstrates that co-cultivation of *Chlorella vulgaris* with N_2_-fixing bacteria under nitrogen-free conditions achieves biomass productivity comparable to nitrogen-supplemented controls, and the resulting culture supernatants significantly improve seed germination indices, with increases exceeding 40% [64]. Such consortia represent a promising strategy to improve soil fertility, increase crop yields, and reduce reliance on synthetic nitrogen fertilizers. However, other reported benefits involve indirect or combined mechanisms. For instance, SLs exuded upon AMF colonization not only recruit fungal partners but also trigger hyphal branching, which expands the root’s absorptive surface; the resulting improvement in phosphorus and water acquisition depends on both exudate-mediated signaling and the physical establishment of the mycorrhizal network [102]. Collectively, these mechanisms contribute to increased nutrient-use efficiency, with the strength and directness of the causal relationship depending on the specific nutrient and microbial partner involved. Please refer to Table 4 for relevant examples of the application advantages of PGPMs mediated metabolic reprogramming.

### 5.4. Toward Sustainable Agricultural Practices

The strategic application of PGPMs to leverage plant metabolic plasticity presents a transformative pathway toward sustainable agriculture. Field studies confirm that inoculation with tailored microbial consortia can increase crop yield, improve soil health, and reduce greenhouse gas emissions [29,103,104]. Integrating PGPMs with management practices such as cover cropping and organic amendments further enriches indigenous beneficial microbiota, supporting long-term ecosystem resilience [105,106]. Moving forward, research should prioritize developing context-specific microbial formulations and crop varieties with enhanced exudation plasticity to optimize plant–microbe synergies across diverse agricultural systems.

## 6. Methodological Approaches and Future Directions

### 6.1. Integrated Multi-Omics: Unraveling Complex Interactions

Advancing from correlation to causation in plant–microbe–metabolite research requires the integration of multi-omics approaches. Metabolomics, especially non-targeted profiling of root exudates, is essential for identifying key metabolic signals triggered by PGPM inoculation [33]. Spatially resolved techniques such as mass spectrometry imaging (MSI) now allow visualization of exudate gradients along root segments, uncovering microhabitat-specific interaction patterns [107]. In parallel, metatranscriptomics of the rhizosphere microbiome links microbial taxonomy to expressed functions, pinpointing which microbial genes are activated in response to plant metabolic changes [108]. Integrating these datasets with plant transcriptomics is crucial for building predictive networks that reveal master regulatory nodes, such as key transcription factors, and the metabolites they govern, thereby moving toward a mechanistic understanding of rhizosphere dialogue [109,110].

Despite their transformative potential, translating multi-omics discoveries to agricultural applications remains challenging due to technical, biological, and translational gaps [111]. Integrating heterogeneous omics data (e.g., transcripts, metabolites) across distinct feature spaces requires sophisticated normalization to distinguish genuine signals from technical artifacts [112]. Multi-omics studies typically yield correlative biomarkers rather than causal mechanisms, necessitating experimental validation [113]. Even when mechanisms are elucidated, laboratory-validated microbial consortia often fail under field conditions due to soil heterogeneity, climate variability, and competition with native microbiomes [114,115,116]. High costs and technical barriers limit accessibility, and research remains concentrated on major crops, leaving critical gaps for understudied species and emerging threats [116,117]. Standardized protocols and biosafety guidelines for product development are urgently needed [115]. Addressing these challenges will require interdisciplinary collaboration integrating plant science, microbiology, bioinformatics, and machine learning, alongside iterative feedback between multi-omics profiling and hypothesis-driven experimentation [113].

### 6.2. Synthetic Microbial Communities: Testing Function in Defined Systems

The inherent complexity of native soil microbiomes often obscures the functional contribution of individual members. Synthetic Microbial Communities (SynComs)—minimal, defined consortia of genetically tractable microbes—offer a powerful reductionist tool to mechanistically test hypotheses regarding metabolic interactions [118]. For instance, SynComs have demonstrated how specific bacterial taxa can restore defensive phenotypes in coumarin-deficient Arabidopsis mutants, providing direct causal evidence for the role of these metabolites in microbiome assembly [57]. Looking forward, a key frontier lies in developing multi-kingdom SynComs that incorporate bacteria, fungi, and protists to better approximate natural community complexity and achieve more stable, resilient outcomes under open-field conditions [119].

### 6.3. Leveraging Plant Genetics and Genome Editing

A promising strategy for sustainable crop improvement involves developing plant varieties with an enhanced innate capacity to recruit beneficial microbiomes through optimized root exudation. Genome-wide association studies (GWAS) are increasingly pinpointing plant genetic loci associated with the assembly of specific rhizosphere microbial communities [120,121]. Complementing this, CRISPR-Cas-mediated genome editing enables precise manipulation of key plant genes involved in recognizing beneficial microbes (e.g., pattern-recognition receptors), transducing their signals (e.g., transcription factors like MYB72), and transporting effector metabolites (e.g., ABC and ALMTs) [122]. Ultimately, this approach aims to design crops that function as selective “microbial traps,” enriching for beneficial PGPMs and suppressive microbiomes through tailored root exudate profiles.

However, genetic engineering methods also have risks that need to be carefully evaluated. Enhanced secretion of specific metabolites imposes significant carbon costs, potentially diverting resources from growth and reproduction and reducing yield [123]. Moreover, altering the root’s chemical profile may inadvertently affect pathogen susceptibility: metabolites that recruit beneficial microbes could also attract or stimulate pathogenic species, disrupting the delicate balance of plant immunity [124,125]. For instance, modifications to defense signaling pathways (e.g., SA, NHP) can trigger unintended autoimmunity or compromise disease resistance [124]. The rhizosphere’s complexity further complicates outcomes, as engineered signals may not reliably recruit target microbes across diverse soil types and environmental conditions [125]. Addressing these risks requires a systems-level understanding of plant-microbe interactions, integrating multi-omics to guide precise, context-aware engineering strategies that balance recruitment benefits against metabolic costs and ecological safety [123,125,126].

### 6.4. Future Directions and Challenges

The large-scale application of PGPMs faces interrelated technical and knowledge-based challenges (Table 5). A primary technical hurdle is the limited shelf life and field persistence of many formulations, as microbial viability is often reduced by environmental stressors such as light, heat, and desiccation. This issue is compounded by the absence of standardized, effective delivery systems and application protocols adapted to diverse soil and climatic conditions, leading to inconsistent field performance [127,128].

PGPM efficacy is highly dependent on dynamic interactions among microbial traits, plant genotype, and environmental conditions, which collectively contribute to the variability and unpredictability of outcomes under field conditions. First, microbial strains exhibit pronounced specificity and adaptive variability: isolates such as the siderophore-producing *Bacillus altitudinis* AS19, which promotes seed germination and plant growth under iron-limited conditions [129], may fail to establish competitive populations in complex field soils where native microbiota outcompete introduced strains [115]. Second, single-strain inoculants often show limited adaptability, whereas microbial consortia can provide greater functional resilience under challenging conditions. For instance, in a low-fertility alkaline sandy soil under open-field drip irrigation, a consortium product outperformed single-strain inoculants in enhancing phosphate uptake and biomass production, whereas all inoculants performed similarly under controlled greenhouse conditions [130]. Third, many PGPM-mediated benefits operate through indirect mechanisms (e.g., siderophore-mediated iron competition, induced systemic resistance) that “manage” rather than fully control pathogens [115], and their expression is conditional on soil nutrient status, pathogen pressure, and other edaphic factors. Consequently, PGPM effects are inherently less predictable and immediate than those of synthetic agrochemicals.

Mechanistically, significant gaps remain. Most mechanistic insights are derived from sterile laboratory systems, leaving the survival, colonization, functional stability, and interactive dynamics of introduced PGPMs within complex native soil communities poorly understood. Moreover, while core regulators such as MYB72 and JA/ET pathways are likely conserved across angiosperms, the specific metabolites are expected to be highly species- and genotype-specific. A major current limitation is that our detailed molecular understanding is largely derived from model species (e.g., *Arabidopsis*, tomato), while the genetic and metabolic complexity of most non-model crops remains largely uncharted.

Ecologically, the establishment and efficacy of inoculants are highly variable across environments, and their long-term impact on resident microbial communities and ecosystem functions requires further study. At the molecular level, the regulatory networks through which PGPMs reprogram plant metabolism and exudation are not fully mapped; specifically, how different PGPMs precisely modulate host transcriptomes and metabolic fluxes to assemble a beneficial microbiome remains unclear. Furthermore, the crosstalk between PGPM-modulated hormonal pathways (e.g., auxin, JA, ET) and their integration in plant stress responses needs deeper exploration. Finally, translating laboratory-identified key genes (e.g., *MYB72*) and regulatory modules into practical crop genetic improvements or robust microbial formulations lacks systematic field validation and cross-scale integrative research frameworks.

**Figure 3:**
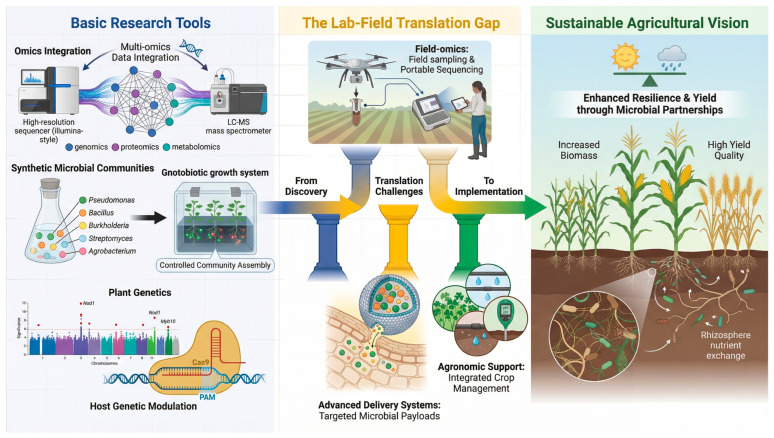
Integrated framework for future research and application of PGPM-mediated metabolic reprogramming. Multi-omics and machine learning identify key microbial strains, plant genes, and metabolites, enabling the design of synthetic communities (SynComs) that are validated under controlled conditions. A critical translational gap is addressed through advanced formulation technologies (e.g., nano-encapsulation) to enhance microbial survival, and genome editing (e.g., CRISPR-Cas) to develop crop varieties with optimized root exudation profiles that selectively recruit beneficial microbes. In the field, inoculated crops are integrated with sustainable agronomic practices and monitored via field-omics to achieve the ultimate goal of resilient rhizosphere microbiomes that enhance productivity, nutrient use efficiency, and stress tolerance while reducing synthetic inputs.

A central translational barrier in PGPM utilization is the frequent failure of laboratory-effective inoculants under field conditions. To bridge this gap, future work should adopt integrated and application-oriented research strategies. First, field-omics approaches—longitudinal multi-omics analyses across varied soils, climates, and management regimes—are needed to identify the environmental and agronomic factors that govern the success of PGPM-induced metabolic reprogramming in real agricultural settings [131]. Second, advanced delivery systems, such as nano-encapsulation and enhanced seed coatings, must be developed to improve the survival, colonization, and metabolic activity of inoculated microbes within competitive soil environments [132]. Third, agronomic practices that foster adapted native microbiomes—including cover cropping, organic amendments, and reduced tillage—should be optimized to support resident beneficial PGPMs, reducing sole reliance on external inoculation [133,134]. By combining reductionist synthetic community tools with field-scale ecological studies, and by leveraging plant genetics, we can progress from describing interactions toward predictably engineering the rhizosphere for more sustainable agriculture (Figure 3).

## 7. Conclusions

The dynamic interplay between plants and PGPMs constitutes a sophisticated form of biological mediation, wherein microbes act as metabolic reprogrammers of the host. As summarized in this review, PGPMs confer benefits that extend beyond direct growth promotion, primarily by triggering metabolic shifts in the plant. This reprogramming results in the secretion of a tailored blend of root exudates—including organic acids, coumarins, strigolactones, and other specialized metabolites. These exudates function as potent ecological filters, selectively shaping microbial community assembly, enriching beneficial taxa, and suppressing pathogens.

The molecular basis of this reprogramming involves core phytohormonal signals-such as JA, ET, and SLs-and key transcription factors, including MYB72, ERF1, and GRAS-family proteins. Together, they orchestrate the expression of metabolic pathways and transporter genes, establishing self-reinforcing feedback between the plant and its microbiome. This interplay amplifies systemic resistance, improves nutrient acquisition, and increases abiotic stress tolerance, ultimately fostering a more resilient and functionally integrated plant holobiont adapted to thrive under suboptimal conditions.

Translating mechanistic insights into reliable field applications represents the next critical frontier. Although integrated multi-omics and SynComs provide powerful tools to unravel this complexity, the strong context-dependency of agricultural environments continues to pose a major translational hurdle. Progress will depend on interdisciplinary strategies that merge microbial ecology with plant genetics—for instance, using genome editing to develop crop varieties with an enhanced innate capacity to recruit beneficial microbes, coupled with advances in formulation science to deliver stable and effective inoculants. However, such genetic interventions must be pursued with caution, acknowledging potential ecological risks (e.g., unintended effects on non-target soil organisms or gene flow to wild relatives) and regulatory constraints that vary across jurisdictions. Rigorous biosafety assessments and context-specific risk evaluation will be essential to ensure responsible deployment. Ultimately, leveraging PGPM-mediated metabolic reprogramming offers a viable route to engineer more resilient rhizospheres, supporting a transition toward productive, sustainable, and climate-adapted agricultural systems underpinned by microbiome management.

## Figures and Tables

**Table 1:** Representative PGPM groups, their functions, and documented case studies.

PGPM Group	Representative Genera	Key Functions	Examples	References
Bacteria	*Pseudomonas*, *Bacillus*, *Rhizobium*, *Azospirillum*, *Burkholderiar*	N_2_ fixation, P solubilization, IAA production, ISR induction	*P. simiae* WCS417 induces ISR in *Arabidopsis* via MYB72; *B. velezensis* FZB42 promotes growth and suppresses pathogens in multiple crops	[36,37]
Fungi (AMF)	*Rhizophagus*, *Funneliformis*, *Glomus*	P and N acquisition, water uptake, soil aggregation	*R. irregularis* enhances P uptake in maize under low-P conditions; AMF colonization improves drought tolerance in wheat	[38,39]
Fungi (Biocontrol)	*Trichoderma* spp.	Mycoparasitism, antibiotic production, ISR induction	*T. harzianum* T-78 triggers ISR against *Botrytis cinerea* in *Arabidopsis* via MYB72 and NO signaling	[40]
Microalgae	*Chlamydomonas*, *Chlorella*, *Scenedesmus*	IAA production, O_2_ provision, biofilm formation	*C. reinhardtii* produces IAA via LAO1, promoting mutualism with *Methylobacterium*; *Chlorella*-bacteria consortia enhance N availability in rice	[30,41]
Cyanobacteria	*Anabaena*, *Nostoc*, *Synechococcus*	N_2_ fixation, EPS production, soil stabilization	*Anabaena* enhanced the length of cucumber seedling stems; cyanobacterial inoculants improve rice yield	[42,43]

**Table 3:** Key Transcriptional Regulators and Transporters in PGPM-Induced Metabolic Reprogramming.

Regulator/Transporter	Type	Induced by	Target Process/Metabolite	Function in Rhizosphere	References
MYB72	Transcription Factor	*Pseudomonas* spp.	Coumarin biosynthesis	Iron chelation, pathogen suppression	[52,67,68]
ERF1	Transcription Factor	JA/ET signaling	Antimicrobial synthesis	Broad-spectrum defense	[69,70,71]
GRAS (e.g., NSP1)	Transcription Factor	AMF	Strigolactone biosynthesis	Hyphal branching, symbiosis	[72,73]
ALMT1	Transporter	PGPM, Low Pi, Acid soil	Malate, Citrate efflux	P solubilization, pH modulation	[77,78,79]
ABC transporters	Transporter	Iron deficiency, PGPR	Coumarin secretion	Microbial recruitment, Fe uptake	[80,81]

**Table 4:** Overview of application advantages of PGPM-induced metabolic reprogramming in agriculture.

Application Domain	Specific Mechanism	Representative PGPMs	Key Plant Metabolites/Processes
Shaping a disease-suppressive microbiome	Selective recruitment of beneficial microbes; direct production of pathogen-inhibiting compounds.	*Pseudomonas* spp. (e.g., *P. simiae*), *Bacillus* spp., *specific fungi*	Benzoxazinoids, phenolic acids, flavonoids, coumarins (e.g., scopoletin)
Enhancing abiotic stress tolerance	Improvement of soil environment (e.g., water retention); direct assistance in stress response (e.g., ion homeostasis, ROS scavenging).	*Bacillus *spp., *Pseudomonas* spp., AMF	Osmolytes (e.g., proline), exopolysaccharides, organic acids, ACC deaminase activity
Improving nutrient acquisition and use efficiency	Chelation of sparingly soluble nutrients; signaling molecules to promote beneficial symbioses.	*Pseudomonas* spp., Rhizobia, AMF, specific microalgae-N_2_-fixing bacteria consortia	Coumarins (e.g., scopoletin), organic acids (e.g., citrate, malate), flavonoids, SLs
Induce plant systemic resistance	ISR via JA/ET signaling pathways	*Pseudomonas* spp. (e.g., *P. simiae*, *P. capeferrum*), *Bacillus* spp. (e.g., *B. amyloliquefaciens*), *Trichoderma* spp.	*MYB72* activation

**Table 5:** Comparison of methodological approaches for studying and engineering PGPM-mediated metabolic reprogramming.

Method	Core Principle	Advantages	Limitations
Multi-omics (genomics, transcriptomics, metabolomics, metatranscriptomics)	High-throughput molecular profiling to identify genes, transcripts, metabolites, and microbial functions	Comprehensive, unbiased, hypothesis-generating; reveals novel pathways and biomarkers	Data heterogeneity across platforms; distinguishing biological signals from technical artifacts; correlation ≠ causation; high cost and expertise required
SynComs	Defined consortia of culturable microbes designed to recapitulate key functions of native microbiomes	Reductionist approach enables causal testing of microbial interactions and functions; reproducible under controlled conditions	May oversimplify natural complexity; community stability uncertain under field conditions; requires extensive isolation and characterization
Genome Editing (CRISPR-Cas)	Precise modification of plant genes involved in signaling (e.g., *MYB72*), transport (e.g., *ABC* transporters), and exudate biosynthesis	Enables causal testing of gene function; potential to create “microbe-recruiting” crop varieties	Off-target effects; regulatory constraints; ecological risks (gene flow, non-target effects); trade-offs (carbon cost, pathogen susceptibility)
Advanced Formulation Technologies (nano-encapsulation, alginate microencapsulation, fluidized bed drying)	Physical protection of microbial cells to enhance survival during storage and after application	Extended shelf-life; protection from desiccation, temperature stress, and predation	Cost; scalability; carrier material biocompatibility; inconsistent field performance across soil types
Field-Omics	Longitudinal multi-omics monitoring under real-world agricultural conditions	Identifies environmental and agronomic factors governing PGPM success; validates lab findings in situ	High complexity; confounding variables; requires extensive replication across sites and seasons

## Data Availability

No new data were created or analyzed in this study. Data sharing is not applicable to this article.
